# Longitudinal associations between different types of screen use and depression and anxiety symptoms in adolescents

**DOI:** 10.3389/fpubh.2023.1101594

**Published:** 2023-04-27

**Authors:** Fatima Mougharbel, Jean-Philippe Chaput, Hugues Sampasa-Kanyinga, Ian Colman, Scott T. Leatherdale, Karen A. Patte, Gary S. Goldfield

**Affiliations:** ^1^School of Population Health, University of Ottawa, Ottawa, ON, Canada; ^2^Healthy Active Living and Obesity Research Group, Children’s Hospital of Eastern Ontario Research Institute, Ottawa, ON, Canada; ^3^School of Epidemiology and Public Health, University of Ottawa, Ottawa, ON, Canada; ^4^Centre for Fertility and Health, Norwegian Institute of Public Health, Oslo, Norway; ^5^School of Public Health Sciences, University of Waterloo, Waterloo, ON, Canada; ^6^Department of Health Sciences, Faculty of Applied Health Sciences, Brock University, St. Catharines, ON, Canada

**Keywords:** screen time, depression, anxiety, adolescents, public health

## Abstract

**Background:**

Evidence examining the longitudinal associations between different types of screen behaviours and mental health among adolescents is limited. The present study examined the association between five types of screen behaviours and symptoms of anxiety and depression one year later. This study also assessed how changes in screen time were associated with changes in anxiety and depressive symptoms and whether the observed relationships were moderated by sex.

**Methods:**

Longitudinal data of 17,174 students in grades 9–12 (53.5% females; mean age: 15.1 ± 0.9 years) attending high schools in Canada from two waves (year 6: 2017/18, year 7: 2018/19) of the COMPASS study were analyzed. Leisure screen time and mental health measures were self-reported. To test if the associations between screen time and anxiety, and depression vary by sex, two-way interactions were examined for sex. Analyses accounted for school clustering, race/ethnicity, sex, age, income, body mass index *z*-score, and previous year anxiety and depression symptoms.

**Results:**

There were significant longitudinal associations between time spent on each type of screen and subsequent anxiety and depression symptoms. The strength of the associations varied by type of screen behaviour. Interaction analysis indicated a sex difference for television viewing and anxiety and depression symptoms, and internet surfing and anxiety symptoms. A dose-response relationship was observed between phone talking and anxiety symptoms. Beta estimates indicated that an increase in screen duration was associated with a further increase in anxiety and depression symptoms.

**Conclusion:**

Higher screen time was longitudinally associated with higher anxiety and depression symptoms at one-year follow-up in adolescents. Time-change associations between screen usage and depressive and anxiety symptoms were observed. Also, associations differed based on sex and screen type, whereby greater increases in screen use predicted greater emotional distress. Findings from this prospective analysis suggest that screen time is an important determinant of anxiety and depressive symptoms among adolescents. Future studies are recommended to help inform programs promoting screen time reduction with a goal to enhance adolescents’ mental health.

## Background

Mental disorders among youth have become a serious public health problem worldwide, with one in seven adolescents aged 10–19 years experiencing mental disorders (1). Up to 70% of mental disorders start before the age of 18 years (2), and many persist into adulthood. Mental disorders are a leading cause of disability and disease burden globally, of which anxiety and depression are the main contributors (3, 4). It has been estimated that anxiety and depression make up 43% of mental disorders among adolescents aged 10–19 years, within which nearly 31.4% of males and 56.3% of females were affected (5). Given the early onset and substantial societal, health, and economic burden of anxiety and depression (6–9), it is critical to examine the modifiable risk factors for the prevention and early intervention of mental disorders.

In recent years, excessive time spent on screens among adolescents has been recognized as a concerning issue associated with mental disorders, including anxiety and depression, especially with the widespread accessibility of digital devices and platforms (10–14). It has been indicated that 95% of United States adolescents (15) and 100% of Canadian adolescents and young adults (16) have access to smartphones, with the majority of them using the internet regularly (15, 17). Canadian representative data (18) show that youth are the greatest leisure users of screens with a daily screen time ranging from 3.1–7.6 h per day, exceeding the Canadian and World Health Organization sedentary behaviour guidelines of two hours or less of daily sedentary recreational screen usage (19, 20). Most adolescents spend more than three hours a day (35% spend five hours daily) on leisure screen time (21). This is very concerning considering the strong associations between screen time and anxiety and depression among adolescents (22–24); however, additional longitudinal evidence is required as suggested by recent reviews (11–14).

It has been suggested that different types of screen behaviours are associated differently with anxiety and depression, though results are not consistent (12–14, 22). For example, television viewing was shown to be more weakly associated with depression than other types of screen use, such as using a computer or video gaming (13, 14, 24). On the other hand, all screen types were found to be associated with anxiety (13). Other results indicated that video gaming and television viewing have stronger associations with depression and anxiety when compared to internet surfing and mobile phone use (12). Given the ongoing changes in screen types and how youth engage in screen time behaviour, it is important to prospectively examine whether each type of screen behaviour is differently or similarly related to depression and anxiety to better inform research, policy and practice.

Previous studies suggest that recreational screen time can vary between males and females. While adolescent males report playing more computer and video games, females report spending more time on social media and chatting online (15, 17, 25, 26). However, results on the moderating role of sex on the association between screen time and anxiety and depression were examined in only a few studies and with mixed results (12, 13). One study found that watching television was negatively associated with depressive symptoms one year later in males and positively associated with depressive symptoms one year later in females (27). Another study indicated that males who played video games the most at baseline had the lowest level of anxiety after one year, though females who played video games the most at baseline had the highest level of anxiety one year later (28). Another study found no sex difference between different types of screen and depressive symptoms (29, 30).

Given limited longitudinal research has examined how changes in different types of screen time could be associated with changes in anxiety and depressive symptoms over time and how sex moderates these associations, there is a critical need for more research in this field, especially that the majority of adolescents exceeding screen time guidelines. Such insight could help inform educational programs and the development of targeted interventions designed to reduce screen time and displace it with more beneficial activities for adolescents’ mental health. Therefore, the objectives of this study are threefold: (1) examining the longitudinal associations between five types of screen time (including communication-based screen time such as instant messaging, chatting, and texting) and symptoms of anxiety and depression among adolescents; (2) assessing how changes in screen time are associated with changes in anxiety and depressive symptoms one year later; and (3) evaluating whether the observed relationships between screen time and symptoms of anxiety and depression are moderated by sex.

## Design

The COMPASS study is a designed to collect hierarchical longitudinal data annually (2012–2027) from a rolling cohort of grade 9 to 12 (Secondary I–V in Quebec) students from a convenience sample of secondary schools in British Columbia, Alberta, Ontario, and Quebec, Canada. School boards and schools were purposefully selected based on whether they permitted active information passive-consent parental permission protocols. All students attending participating schools and not withdrawn by their parents were eligible to participate. All participating students provided assent at the time of data collection. During the waves of data used here, all data were self-reported using a paper-and-pencil questionnaire completed during class time. All COMPASS procedures were approved by the University of Waterloo Office of Research Ethics (ORE #: 30118) and appropriate school board committees. A full description of the COMPASS host study is available elsewhere (31).

## Participants

For the current study, we used linked student-level data from year 6 (2017/18; Y6—time 1) and year 7 (2018/19; Y7—time 2) of the COMPASS host study.^1^ A one-year follow up was chosen because year 6 (2016/17) was the first year that the mental health measures were included in all participating schools and year 8 (2019/20) was interrupted by the start of the COVID-19 pandemic school closures.

In year 6, 66,501 students from 117 secondary schools participated, with a participation rate of 81.9%. In year 7, 74,501 students from 136 secondary schools participated in the study, with a participation rate of 84.2%. Each school was assigned a unique identifier, and student data were linked through a unique, self-generated anonymous code (32). Non-linkage was primarily due to student absences on one of the two data collection dates for reasons related to scheduled study periods, graduation, school transferring, dropping out of school, school field trips, and sports events, or for inaccurate data given on the data linkage measures. Further information on the COMPASS design is available elsewhere (31).

Of the 23,557 grade 9 to 12 students who were successfully linked for their participation in COMPASS in year 6 [considered here as time 1 (T1)] and year 7 [considered here as time 2 (T2)], 17,310 had complete data regarding depressive and anxiety symptoms at both time points. Among them, 17,147 students had complete data for all variables included in our analyses at both time points and constituted our analytical sample.

## Measures

### Screen time

Screen time was measured by asking the participants the following question: “how much time per day do you usually spend doing the following activities?,” where the five items of interest were: (1) “watching/streaming television shows or movies”; (2) “playing video/computer games”; (3) “talking on the phone”; (4) “surfing the internet”; and (5) “texting, messaging, emailing.” Individuals reported their screen time in hours (ranging from 0 to 9) and minutes (ranging from 0 to 45) for each item. This measure has been validated for use among adolescents (33). To assess a possible dose-response gradient and allow for good distribution across categories, we categorized each item in 5 categories as follows: [0 to 29 min/d (coded 0—reference category); 30 to 59 min/d (coded 1), 1 to <2 h/d (coded 2); 2 to <3 h/d (coded 3); ≥ 3 h/d (coded 4)].

### Anxiety

General anxiety symptoms were measured using the generalized anxiety disorder 7-item scale (GAD-7), a tool widely used in both clinical practice and research, which has been validated for use in youth (34, 35). The self-reported scale assesses generalized anxiety symptoms that include difficulty controlling feelings of worry, feeling afraid, trouble relaxing, nervousness, irritability, and restlessness over the last 2 weeks. Response options were as follows: “not at all,” “several days,” “over half the days,” or “nearly every day.” Responses were scored from 0 to 3 and summed to calculate a total anxiety score that ranged from 0 to 21, with higher scores indicating higher levels of anxiety symptoms. For the purpose of the present study, two forms of this variable were used in the analysis: (1) the total score was used as a continuous variable, and, (2) a dichotomous variable with a binary coding system to categorize participants with (coded 1) and without (coded 0—reference category) clinically-relevant anxiety symptoms with a cut-off score ≥10 was used (36).

### Depression

Depressive symptoms were measured using the Center for Epidemiologic Studies Depression Scale (Revised)—10 (CESD-R-10) (37). This self-report scale was designed to assess clinical depressive symptoms that include feelings of sadness, hopelessness, amotivation, difficulty concentrating, difficulty sleeping, and irritability within the last seven days. Response options were as follows: “none or less than 1 day,” “1–2 days,” “3–4 days,” or “5–7 days.” Responses were scored from 0 to 3 and summed to calculate a total score that ranged from 0 to 30, with higher scores indicating higher levels of depression symptoms. The CESD-R-10 scale has demonstrated validity among adolescents and adults (37–39). Two forms of this variable were used in the analysis. The total score was used as both a continuous variable and as a dichotomous variable with a binary coding system to categorize participants with (coded 1) and without (coded 0—reference category) clinically-relevant depressive symptoms using a cut-off score ≥10 (37).

### Covariates

Individual-level covariates included age (years), race/ethnicity, body mass index [BMI; age and sex adjusted BMI based on the WHO classifications using participant reported height and weight (40)], school-area median average household income, previous year anxiety and depressive symptoms, and sex. Race/ethnicity was assessed through self-identification and categorized as follows: White, Black, Asian, Hispanic/Latin American, and Other. BMI was categorized as follows: underweight (coded 1), normal weight (coded 0—reference category), overweight (coded 2), and obesity (coded 3). Missing BMI was included as a category, given the high frequency of nonresponse to height/weight that cannot be excluded as missing completely at random (41) (coded 4). Weight and height measures in COMPASS were validated (42). Average household income was used after cross-referencing school postal codes with Statistics Canada data. Income categories were as follows: $25,000–50,000; $50,001–75,000; $75,001–100,000 and >$100,000. Sex was identified based on the answer to the question “Are you female or male?” Only two response options were provided in these years of the survey: “female” (coded 0—reference category) and “male” (coded 1).

### Change in screen time and anxiety and depression symptoms

Change in screen time was determined by subtracting the time spent on each type of screen at T1 from the time spent at T2. Possible scores ranged from −5 to 5, with a positive score indicating that an individual increased their screen time at T2 compared to T1, and a negative score indicating that an individual decreased their screen time at T2 compared to T1. Scores were treated as scale variables.

Change in symptoms was determined by subtracting anxiety and depression scores at T1 from anxiety and depression scores at T2. Scores ranged from −21 to 21 for change in anxiety scores, and from −26 to 30 for change in depression scores. Positive scores indicate an increase in symptoms and negative scores indicate an improvement in symptoms. Scores were treated as continuous variables.

### Data processing and analyses

Analyses included complete information on all variables (*N* = 17,147). Pearson’s chi-square tests and adjusted Wald tests were used for categorical and continuous variables, respectively, to test the statistical differences between missing data and those included in our analyses for all the variables. Compared to the included participants, those who were excluded were more likely to be male, aged 15-to-20-years, and more likely to use screen more frequently at both T1 and T2, and more likely to report clinically relevant anxiety symptoms at T1 and depression symptoms at T2.

Descriptive statistics, including frequencies, means, and standard deviations, were used to characterize the sample. Mixed models were used to account for school clustering by adding random intercepts at the school level. Odds ratios and 95% confidence intervals (CI) were used to estimate the longitudinal associations between daily time spent on each type of screen and anxiety and depression symptoms. We computed the two-way interactions (sex by each type of screen) on the association between screen time and anxiety and depression. A significant two-way interaction (*p* ≤ 0.05) would indicate significant moderation and models were stratified accordingly. Data are presented based on unadjusted models and models adjusted for year 6 covariates, including anxiety and depression symptoms, and sex when models were not stratified. Given that sex interactions with television viewing and anxiety and depression and internet surfing with anxiety were statistically significant, analyses for these associations were stratified by sex. A sensitivity analysis was conducted using the total anxiety and depression scores (continuous) and the pattern of results was similar (data not shown). Therefore, only analyses with dichotomous anxiety and depression scores are presented for clarity.

Conditional change models were conducted to examine whether longitudinal changes in daily time spent on each type of screen predict changes in anxiety and depressive symptoms at follow-up. The beta coefficients for change in each type of screen were presented to describe how change in screen time was associated with changes in anxiety and depressive symptoms. Models were adjusted for Y6 covariates, anxiety and depression, and sex when models were not stratified. A sensitivity analysis was conducted using the continuous screen time variable form, and results were similar (data not shown). All analyses were carried out in STATA/SE 16.1.

## Results

### Study sample

This study included a total of 17,174 (53.5% females) participants with a mean age of 15.1 years at T1. Characteristics of the participants at T1 and T2 are summarized in Table 1. Females reported more internet surfing per day than any other screen time behaviour at T1 (mean = 126 min) and T2 (mean = 130 min). On the other hand, males reported more time playing video games at T1 (mean = 142 min) and T2 (mean = 130) than any other screen time behaviour. Concerning mental health characteristics, 24.4% of the sample indicated having clinically-relevant anxiety symptoms (cut-off score ≥10) and 34.2% indicated having clinically-relevant depressive symptoms (cut-off score ≥10). Females reported more clinically relevant anxiety and depressive symptoms compared to males.

**Table 1:** Descriptive characteristics among Canadian secondary school students that participated in year 6 (time 1: 2017–18) and year 7 (time 2: 2018–19) of the COMPASS study.

	Time 1			Time 2		
Characteristics	Total population	Females	Males	Total population	Females	Males
	*N* = 17,174	*N* = 9,173	*N* = 7,974	*N* = 17,174	*N* = 9,173	*N* = 7,974
Age (mean (SD))	15.1 (0.9)	15.1 (0.9)	15.1 (0.9)	16.1 (0.9)	16.1 (0.9)	16.1 (0.9)
Race/ethnicity (%)*						
White	68.3	68.0	68.6	68.3	68.9	68.2
Black	02.5	02.3	02.8	02.5	02.4	03.2
Asian	12.6	12.8	12.6	12.6	12.9	12.5
Latin American/Hispanic	02.8	02.9	02.7	02.8	02.9	03.1
Other and mixed	13.8	14.0	13.3	13.8	12.9	13.0
BMI classification*						
Underweight	01.8	01.5	01.9	01.6	01.2	02.1
Normal weight	57.1	60.0	53.7	60.4	63.3	56.8
Overweight	12.5	11.0	14.3	13.2	12.3	14.3
Obesity	05.6	04.0	07.6	06.1	04.4	08.1
Missing BMI	23.0	23.5	22.5	18.7	18.8	18.7
School-area household median income*						
$ 25,000–50,000	12.6	13.3	11.6	12.6	13.3	11.6
$50,001–75,000	55.8	55.1	56.6	55.8	55.1	56.7
$75,001–100,000	26.6	26.4	26.9	26.6	26.5	26.9
> $ 100,000	05.0	05.2	04.9	05.0	05.2	04.8
*Screen time (mean (SD))*						
Television*	112.36 (87.04)	121.46 (87.74)	101.88 (85.04)	113.47 (87.26)	117.63 (86.25)	108.69 (88.17)
Video games*	81.43 (109.31)	29.16 (64.94)	141.56 (118.68)	74.55 (105.20)	26.48 (61.05)	129.93 (117.45)
Talking on the phone*	38.02 (75.57)	45.71 (81.62)	29.18 (66.87)	44.15 (80.39)	50.14 (84.11)	37.25 (75.30)
Internet surfing*	112.37 (111.98)	126.25 (116.56)	96.41 (104.22)	118.74 (108.29)	130.32 (110.18)	105.39 (104.50)
Texting/messaging/emailing*	94.20 (111.84)	110.38 (119.32)	75.58 (99.37)	99.32 (109.89)	111.98 (114.07)	84.73 (102.96)
*Screen time*						
Television viewing*						
0–29 min/d	12.6	09.2	16.6	12.0	09.5	15.0
30–59 min/d	09.1	09.5	08.7	09.4	10.4	08.3
1–2 h/d	28.3	26.7	30.0	27.2	27.0	27.3
2–3 h/d	25.4	26.4	24.2	26.6	26.9	26.2
3+ h/d	24.6	28.2	20.5	24.8	26.2	23.2
Video games*						
0–29 min/d	45.4	72.2	14.6	47.8	74.2	17.5
30–59 min/d	07.3	07.5	07.0	07.4	07.3	07.5
1–2 h/d	15.5	10.8	20.9	15.8	10.0	22.6
2–3 h/d	12.2	04.6	20.9	11.4	04.0	19.7
3+ h/d	19.6	04.9	36.6	17.6	04.5	32.7
Talking on the phone*						
0–29 min/d	70.0	62.0	76.0	62.9	58.0	68.6
30–59 min/d	08.0	09.2	06.5	08.8	10.0	07.6
1–2 h/d	11.0	14.3	09.5	14.0	15.5	12.4
2–3 h/d	05.0	06.5	04.0	06.7	08.0	05.2
3+ h/d	06.0	08.0	05.0	07.6	08.5	06.2
Internet surfing*						
0–29 min/d	19.5	16.0	23.7	15.0	12.2	18.2
30–59 min/d	13.0	11.7	14.0	12.0	10.8	13.3
1–2 h/d	25.5	24.3	27.0	26.3	24.8	28.0
2–3 h/d	17.0	18.0	16.0	19.4	20.5	18.3
3+ h/d	25.0	30.0	19.3	27.3	31.7	22.2
Texting/messaging/emailing*						
0–29 min/d	29.7	22.0	38.5	24.5	18.5	31.5
30–59 min/d	16.3	17.0	15.5	16.0	16.4	15.5
1–2 h/d	21.5	22.6	20.2	24.2	24.3	24.0
2–3 h/d	13.0	14.4	11.4	14.7	16.7	12.5
3+ h/d	19.5	24.0	14.4	20.6	24.1	16.5
Anxiety symptoms score* (mean (SD))	6.27 (5.53)	7.77 (5.73)	4.55 (4.74)	6.82 (5.67)	8.42 (5.73)	4.99 (5.02)
Anxiety symptoms*						
Yes	24.4	33.5	14.0	27.5	36.9	16.7
No	75.6	66.5	86.0	72.5	63.1	83.3
Depressive symptoms score* (mean (SD))	8.45 (5.84)	9.67 (6.23)	7.05 (5.01)	9.20 (6.04)	10.47 (6.27)	7.74 (5.40)
Depressive symptoms*						
Yes	34.2	42.9	24.2	40.0	49.6	29.4
No	65.8	57.1	75.8	60.0	50.4	70.6

Data are shown as percentages or mean (standard deviation). SD, standard deviation; BMI, body mass index. Starred variables represent statistically significant value of *p*-values for the sex differences (<0.05). Anxiety and depression symptoms assessed by the GAD-7 and CESD-10-R, respectively, with scores ≥10 used to indicate the presence of clinically-relevant symptoms.

### Longitudinal association between time spent on each type of screen and symptoms of anxiety

Odds ratios for the association between time spent watching television/movies, playing video games, talking on the phone, surfing the internet, and screen-based communication and anxiety are presented in Table 2. After adjusting for covariates and previous year anxiety, we found a positive association between time spent playing video games and anxiety (OR for 1–2 h = 1.13, 95% CI: 1.00 to 1.28; OR for 3+ hours = 1.27, 95% CI 1.12 to 1.45). In addition, we found a dose-response association between time spent talking on the phone and anxiety (OR for 30 min—less than 1 h = 1.22, 95% CI: 1.06 to 1.40; OR for 1—less than 2 h = 1.18, 95% CI: 1.05 to 1.33; OR for 2 to <3 h = 1.25, 95% CI 1.06 to 1.48, OR for 3+ hours = 1.36, 95% CI 1.17 to 1.57). Also, communication-based screen time was associated with anxiety symptoms (OR for 2 to <3 h = 1.18, 95% CI 1.04 to 1.34, 3+ hours = 1.40, 95% CI 1.25 to 1.57). Regarding sex differences, we found an association between watching television /movies for three or more hours and anxiety symptoms for females (OR for 3+ hours = 1.27, 95% CI 1.06 to 1.53) but not in males. Surfing the internet for three or more hours was also associated with anxiety symptoms among females (OR for 3+ hours = 1.20, 95% CI 1.03 to 1.40). Higher odds ratios were found among males who/surfed the internet for one to two hours or three or more (OR for 1–2 h = 1.41, 95% CI: 1.17 to 1.69; OR for 3+ hours = 1.49, 95% CI 1.22 to 1.82).

**Table 2:** Longitudinal association between types of screen at time 1 (2017–18) and anxiety symptoms at time 2 (2018–19) among adolescents in the COMPASS study.

		Anxiety symptoms at time 2	
		Crude OR (95% CI)	Adjusted OR (95% CI)
Television viewing			
Television viewing-females	0–29 min/d	1	1
	30–59 min/d	0.99 (0.81–1.22)	0.99 (0.79–1.25)
	1–2 h/d	1.08 (0.91–1.28)	1.10 (0.92–1.33)
	2–3 h/d	1.18 (0.99–1.39)	1.16 (0.96–1.40)
	3+ h/d	1.42 (1.20–1.67) ***	1.27 (1.06–1.53)**
Television viewing-males	0–29 min/d	1	1
	30–59 min/d	0.76 (0.58–0.99)*	0.80 (0.60–1.06)
	1–2 h/d	0.84 (0.70–1.01)	0.83 (0.68–1.01)
	2–3 h/d	1.05 (0.87–1.27)	0.98 (0.80–1.20)
	3+ h/d	1.13 (0.93–1.37)	1.00 (0.81–1.23)
Video games	0–29 min/d	1	1
	30–59 min/d	0.65 (0.57–0.75)***	0.86 (0.73–1.01)
	1–2 h/d	0.68 (0.62–0.75)***	1.13 (1.00–1.28)*
	2–3 h/d	0.54 (0.48–0.60)***	1.14 (0.99–1.32)
	3+ h/d	0.57 (0.52–0.63)***	1.27 (1.12–1.45)***
Talking on the phone	0–29 min/d	1	1
	30–59 min/d	1.42 (1.24–1.61)***	1.22 (1.06–1.40)**
	1–2 h/d	1.49 (1.34–1.65)***	1.18 (1.05–1.33)**
	2–3 h/d	1.62 (1.40–1.87)***	1.25 (1.06–1.48)**
	3+ h/d	1.91 (1.67–2.17)***	1.36 (1.17–1.57)***
Internet surfing			
Internet surfing-females	0–29 min/d	1	1
	30–59 min/d	0.91 (0.76–1.08)	0.94 (0.78–1.14)
	1–2 h/d	1.06 (0.92–1.23)	1.03 (0.88–1.20)
	2–3 h/d	1.12 (0.96–1.30)	1.04 (0.88–1.24)
	3+ h/d	1.42 (1.24–1.63)***	1.20 (1.03–1.40)*
Internet surfing-males	0–29 min/d	**1**	**1**
	30–59 min/d	1.01 (0.81–1.26)	1.00 (0.79–1.27)
	1–2 h/d	1.46 (1.23–1.74)***	1.41 (1.17–1.69)***
	2–3 h/d	1.41 (1.15–1.72)***	1.23 (0.99–1.29)
	3+ h/d	1.79 (1.49–2.15)***	1.49 (1.22–1.82)***
Texting/messaging/emailing	0–29 min/d	1	1
	30–59 min/d	1.29 (1.15–1.43)***	1.07 (0.95–1.21)
	1–2 h/d	1.29 (1.16–1.42)***	1.01 (0.90–1.13)
	2–3 h/d	1.58 (1.42–1.77)***	1.18 (1.04–1.34)**
	3+ h/d	2.21 (2.01–2.44)***	1.40 (1.25–1.57)***

Bold values represent statistically significant results. OR, odds ratio; CI, confidence interval. Models are adjusted for school clustering, age, race/ethnicity, BMI *z*-score, income, and prior year anxiety. *p < 0.05; **p < 0.01; ***p < 0.001. Data from Canadian secondary school students linked across year 6 (2017–18) and 7 (2018–19) of the COMPASS study.

### Longitudinal association between time spent on each type of screen and symptoms of depression

Odds ratios for the association between watching television/movies, playing video games, talking on the phone, surfing the internet, and screen-based communication and depression after one year are presented in Table 3. After adjusting for covariates and previous year depression, we found a positive association between time spent playing video games and subsequent depression (OR for 1–2 h = 1.14, 95% CI: 1.02 to 1.28; OR for 3+ hours = 1.29, 95% CI 1.15 to 1.46). As for anxiety there was evidence of an association between internet use and subsequent depression (OR 3+ hours = 1.24, 95% CI: 1.11 to 1.38). In addition, our results indicated an association between time spent talking on the phone (OR for 3+ hours = 1.33, 95% CI 1.15 to 1.53) and communication-based screen time and symptoms of subsequent depression (OR for 3+ hours = 1.32, 95% CI 1.19 to 1.46). Sex differences were also found in the association between television viewing and subsequent depression. Females who spent 3 or more hours watching television a day had symptoms of depression one year later (OR for 3+ hours = 1.30, 95% CI 1.09 to 1.55) but no association was found among males.

**Table 3:** Longitudinal association between each type of screen at time 1 (2017–18) and depressive symptoms at time 2 (2018–19) among adolescents in the COMPASS study.

		Depressive symptoms at time 2	
		Crude OR (95% CI)	Adjusted OR (95% CI)
Television viewing-females	0–29 min/d	1	1
	30–59 min/d	0.94 (0.78–1.14)	1.03 (0.83–1.28)
	1–2 h/d	1.02 (0.87–1.20)	1.12 (0.94–1.34)
	2–3 h/d	1.11 (0.95–1.31)	1.15 (0.96–1.38)
	3+ h/d	1.44 (1.23–1.69)***	1.30 (1.09–1.55)**
Television viewing-males	0–29 min/d	1	1
	30–59 min/d	0.79 (0.64–0.97)*	0.82 (0.66–1.03)
	1–2 h/d	0.83 (0.72–0.97)*	0.81 (0.68–0.95)
	2–3 h/d	0.91 (0.78–1.07)	0.87 (0.73–1.03)
	3+ h/d	1.06 (0.91–1.24)	0.91 (0.77–1.08)
Video games	0–29 min/d	1	1
	30–59 min/d	0.78 (0.69–0.88)***	1.06 (0.91–1.22)
	1–2 h/d	0.75 (0.69–0.82)***	1.14 (1.02–1.28)*
	2–3 h/d	0.62 (0.56–0.69)***	1.11 (0.98–1.27)
	3+ h/d	0.72 (0.66–0.78)***	1.29 (1.15–1.46)***
Talking on the phone	0–29 min/d	1	1
	30–59 min/d	1.34 (1.19–1.50)***	1.13 (0.99–1.29)
	1–2 h/d	1.37 (1.25–1.51)***	1.07 (0.96–1.20)
	2–3 h/d	1.56 (1.36–1.79)***	1.16 (0.99–1.36)
	3+ h/d	1.97 (1.74–2.24)***	1.33 (1.15–1.53)***
Internet surfing	0–29 min/d	1	1
	30–59 min/d	0.97 (0.86–1.09)	0.90 (0.79–1.03)
	1–2 h/d	1.18 (1.08–1.30)***	1.00 (0.90–1.11)
	2–3 h/d	1.35 (1.22–1.50)***	1.03 (0.91–1.16)
	3+ h/d	1.92 (1.75–2.11)***	1.24 (1.11–1.38)***
Texting/messaging/emailing	0–29 min/d	1	1
	30–59 min/d	1.21 (1.10–1.33)***	1.07 (0.96–1.20)
	1–2 h/d	1.28 (1.17–1.40)***	1.07 (0.96–1.18)
	2–3 h/d	1.45 (1.31–1.61)***	1.11 (0.99–1.25)
	3+ h/d	1.99 (1.82–2.18)***	1.32 (1.19–1.46)***

Bold values represent statistically significant results. OR, odds ratio; CI, confidence interval. Models are adjusted for school clustering, age, race/ethnicity, BMI *z*-score, income, and prior year depression. *p < 0.05; **p < 0.01; ***p < 0.001.

### Longitudinal association between change in time spent on screen-based behaviour and changes in anxiety and depressive symptoms at follow-up

Table 4 summarizes the results of analyses examining the longitudinal association between changes in time spent on screen-based pursuits and change in subsequent anxiety and depressive symptoms, adjusting for all covariates and prior year anxiety and depression scores. Beta estimates showed a positive association between changes in screen duration for talking on the phone and screen-based communication with subsequent anxiety; no association was found for time change in video games and subsequent anxiety. Also, a positive association was found between increase in screen duration for video gaming, talking on the phone, internet surfing, and screen-based communication and increase in subsequent depressive symptoms for the whole sample. That is, students who increased their screen usage at T2 compared to T1 had higher anxiety and depressive symptoms and those who reduced their screen usage had lower anxiety and depressive symptoms at follow-up. Regarding sex differences, increase in television usage was associated with further increase in subsequent anxiety symptoms for females only and in subsequent depressive symptoms for males only. Duration increase in television usage was associated with increase in subsequent anxiety symptoms for females but not for males, and with increase in subsequent depressive symptoms for males and not for females. Furthermore, increase duration in internet usage was associated with more increase in subsequent anxiety symptoms for females than males.

**Table 4:** Association between one-year change in screen time and changes in anxiety and depressive symptoms among adolescents in the COMPASS study (2017–18 to 2018–19).

Screen types	Anxiety symptoms			
	Est.	SE	95% CI	*p*-value
Television viewing				
Television viewing-females	**0.046**	**0.017**	**(0.011–0.080)**	**0.008**
Television viewing-males	0.018	0.017	(−0.015–0.052)	0.269
Video games	0.017	0.014	(−0.010–0.045)	0.216
Talking on the phone	**0.087**	**0.017**	**(0.051–0.119)**	**0.000**
Internet surfing				
Internet surfing-females	**0.071**	**0.016**	**(0.039–0.102)**	**0.000**
Internet surfing-males	**0.053**	**0.016**	**(0.019–0.086)**	**0.001**
Texting/messaging/emailing	**0.067**	**0.012**	**(0.043–0.092)**	**0.000**
**Screen types**	**Depressive symptoms**			
	**Est.**	**SE**	**95% CI**	***p*-value**
Television viewing				
Television viewing-females	0.030	0.019	(−0.006–0.068)	0.109
Television viewing-males	**0.068**	**0.018**	**(0.032–0.104)**	**0.000**
Video games	**0.041**	**0.015**	**(0.011–0.071)**	**0.007**
Talking on the phone	**0.084**	**0.017**	**(0.050–0.118)**	**0.000**
Internet surfing	**0.070**	**0.012**	**(0.045–0.095)**	**0.000**
Texting/messaging/emailing	**0.100**	**0.013**	**(0.071–0.124)**	**0.000**

Bold values represent statistically significant results. Models are adjusted for school clustering, age, race/ethnicity, BMI *z*-score, income, prior year anxiety, and depressive symptoms. Est., beta coefficient; SE, standard error, CI, confidence interval.

## Discussion

We found that higher screen use is associated with increases in symptoms of depression and anxiety one year later among a large linked-longitudinal sample of Canadian adolescents. Dose-response relationships were observed with talking on the phone and anxiety symptoms and sex differences were observed with television/movie viewing and both anxiety and depression and with internet surfing and anxiety. The association between screen usage and mental disorders varied by the time spent in various screen behaviours and the type of screen behaviour, especially with anxiety. Lastly, our conditional change models indicated a positive linear association between change in the time spent on screens and symptoms of anxiety and depression one year later.

We found that all types of screen behaviours were associated with higher levels of anxiety and depression longitudinally among youth in our sample. While the effect sizes were small, adjusting for the previous year’s anxiety and depression, which is considered an analytical strength, likely attenuated the association and the dose-response pattern. In addition, when escalating the small effect to the population level, we believe the burden increase suggests a high importance of the results. Our results may be explained, in part, by the displacement theory (43, 44). While some evidence in the literature suggested that a moderate level of screen use is not harmful, high screen usage could displace time spent engaging in other beneficial pursuits, such as physical activity, sleep, and in person interaction, which are known to reduce anxiety and depression symptoms (45–49). Many movement guidelines for children and youth, including the Canadian 24-h movement guidelines, discussed the importance of limiting recreational sedentary screen behaviours to no more than 2 h per day, accumulating at least 60 min of moderate to vigorous physical activity daily, and sleeping 9–11 h per night (for those aged 5–13 years) or 8–10 h per night (for those aged 14–17 years) (19, 20). When doing an extensive amount of screen pursuits daily, it is more likely that this time interferes with other important health behaviours (30, 50, 51).

Although, we did not analyze how physical activity, screen time, and sleep, all combined are associated with mental health, previous studies showed that meeting all three recommendations was associated with lower odds of mental health problems (52, 53).

In addition to that, a systematic review (54) suggested that independent of physical activity levels, screen time-based sedentary behaviours are associated with increased psychological problems. Therefore, to maximize mental health benefits, a holistic approach that includes but not limited to screen time reduction is recommended.

In line with previous work (12, 13), we found that the strength of the association between screen time and mental disorders varied by the type of screen used. These findings were more evident for anxiety symptoms. Screen-based communication and talking on the phone were associated with the highest levels of anxiety and depressive symptoms, followed by video gaming. While mixed results were reported in the literature, a recent systematic review of the association between screen time and mental health found little evidence of a positive association between television viewing or videogaming compared to internet use (12). Evidently, the relationship between screen use and depression and anxiety is complex, with many contributing factors and mechanisms. For example, in addition to the displacement hypothesis, talking on the phone and using screen-based communication for an extended period of time could affect mental health by disrupting sleep. It has been estimated that 36% of teens wake up at least once at night to check their phones (55), with the majority of adolescents bringing their phone to their bedrooms (56), which can delay their sleep time (57). Lack of sleep and poor quality sleep are associated with anxiety and depressive symptoms (58).

We also found that spending excessive time on video games was associated with anxiety and depressive symptoms. The effects of videogaming should be approached in regards of several interacting dimensions such as the age of the player, the time spent, personality attributes, and whether gaming alone or with friends (59). Evidence suggest that excessive video gaming, especially playing games that contain violence, is associated with an alteration in frontolimbic and subcortical regions of the brain that are connected to emotion regulation (60–62). This alteration occurred with responses to emotional stimuli that are exaggerated in the amygdala and known to be associated with symptoms of anxiety and depression (63–65).

Our results revealed several differences between sexes. Females spent more time surfing the internet but the association between surfing the internet and anxiety indicates that females had fewer anxiety symptoms than males. The association between frequent use of internet use and anxiety among females could be related to the types and platforms used online. For example, it had been well known that females spend more time using social media platforms, and online chatting which can be beneficial to connect with friends when used moderately and mindfully (15, 66). However, excessive time spent on social networking sites and the passive use of these sites is known to be associated with more exposure to unrealistic content and posts (67, 68) that could lead to upward social comparisons, and envy which in turn can trigger internalizing problems such as anxiety and depression (69–72). On the other hand, sex differences in the strength of the association between time spent on the internet and subsequent anxiety symptoms could be explained by content preferences and how the internet was used (e.g., passive vs. active use). A recent study (73) examined teen’s behaviour on the web and found sex differences regarding internet behaviour, where females are more inclined towards communication and information and show more awareness and caution regarding risky behaviours. This was also confirmed elsewhere (74, 75). Males tend to be less cautious when surfing the web, which may lead to more risky situations (73) such as gambling (76, 77), pornography, and sexting (78). These behaviours may increase their vulnerability to mental disorders like depression and anxiety (78, 79). Cyberbullying was indicated as another mechanism that have particularly negative impacts on anxiety and depression (80–82). Some studies also suggest that higher rates of cyberbullying were found among males (83, 84).

A sex difference was also found in the association between television usage and depression and anxiety symptoms. We found that only females who watched television excessively experienced increased levels of depression and anxiety one year later. As with social media, some television shows or media could expose adolescents to ideal images that promote upward social comparison through ideal body image advertisements (85), another factor contributing to depression and anxiety (86, 87).

Recent studies (12–14) reported inconsistent evidence of sex moderating the association between total screen time or different types of screen use and both depressive symptoms and anxiety among adolescents. This inconsistency could be related to the content of the screen and how it is being used (active vs. passive), which was not well explored in previous work. Since few studies beside the present one analyzed the longitudinal effect by sex, it is hard to draw a conclusion regarding sex differences. Given that the content, quality, and nature of screen use and the way they are used were not measured in the survey, it is critical to consider additional measures in the future to better understand the underlying factors for the observed results, especially sex differences.

Lastly, regarding results from conditional change analyses, the current study is among the first to longitudinally examine conditional change in various types of screen time and anxiety and depression by sex. Our results suggest that increased time spent talking on the phone and screen-based communication were associated with increased depressive and anxiety symptoms. Also, increased in time spent on video games was associated with increased levels of depression with no significant results for anxiety. More time spent on television viewing was associated with higher anxiety symptoms among females and higher depressive symptoms among males. A similar association was observed between the time change in internet surfing, and symptoms of anxiety and depression with higher levels of anxiety found among females. While it is hard to draw a conclusion from these results, more research is warranted to explore these associations in more depth.

Our study includes many strengths. The longitudinal study design and measures of time spent using five different types of screens in a large sample of adolescents add a unique contribution to the literature. We also examined the moderating role of sex on the associations and included both anxiety and depressive symptoms. All measures of screen time and mental health have demonstrated validity in adolescents, including within the COMPASS host study. A significant strength is the adjustment for previous anxiety and depressive symptoms, given that the majority of studies on this topic were cross-sectional. Furthermore, our study contributes to the literature by using a rigorous conditional change analysis model which better establishes directionality given that most previous studies simply examined the longitudinal association between screen time and mental health fixed at each time point.

Although our study provides important insights, there are some limitations that could inform future research. First, we could not examine the screen use content, context, and motives, which are important to determine why various types of screens affect mental health differently and why sex differences occur. Examining those factors can help to maximize the benefits and opportunities adolescents can get from technology and protect them from risks that could occur with certain context when using the screen excessively. Second, although we controlled for baseline levels of anxiety and depression in all models, the possibility of residual confounding may have influenced results. Finally, future studies should rely on longer follow-up periods and assess a broader spectrum of mental health indicators such as, emotional, psychosocial, and cognitive health.

## Conclusion

With high daily screen time exposure reported in the present study, our findings provide longitudinal evidence in a population-based sample of adolescents that more time spent on screens is associated with more anxiety and depressive symptoms. The relationship between some types of screens such as talking on the phone and anxiety appears to follow a dose-response pattern. Sex differences in longitudinal associations were observed between types of screen use (i.e., television viewing and internet surfing) and anxiety and depression. Moreover, our results established that greater increases in screen time were associated with greater increases in depression and anxiety symptoms, and these observations occurred over one year and when controlling for baseline levels of anxiety and depression. More studies are warranted to examine the content and nature of screen usage. These studies can help inform public health programs, tailored with sex-specific and screen-specific considerations in mind, focusing on reducing screen time could contribute to the prevention of anxiety and depression among adolescents.

## Data availability statement

The data analyzed in this study is subject to the following licenses/restrictions: COMPASS study data is available upon request through completion and approval of an online form: https://uwaterloo.ca/compass-system/information-researchers/data-usage-application. Requests to access these datasets should be directed to https://uwaterloo.ca/compass-system/.

## Ethics statement

The studies involving human participants were reviewed and approved by University of Waterloo (ORE#30118), Brock University (REB#18-099), CIUSSS de la Capitale-Nationale-Université Laval (#MP-13-2017-1264), and participating school boards, including the use of active-information passive-consent parental permission protocols. Written informed consent to participate in this study was provided by the participants’ legal guardian/next of kin.

## Author contributions

FM, J-PC, and GG participated in the conception of the study. FM and HS-K conducted statistical analyses. FM wrote the first version of the manuscript. J-PC and GG substantially contributed to the methods and interpretation of results. J-PC, GG, HS-K, IC, SL, and KP critically reviewed the manuscript. All authors contributed to the article and approved the submitted version.

## Funding

The COMPASS study has been supported by a bridge grant from the CIHR Institute of Nutrition, Metabolism and Diabetes (INMD) through the “Obesity—Interventions to Prevent or Treat” priority funding awards (OOP-110788; awarded to SL), an operating grant from the CIHR Institute of Population and Public Health (IPPH) (MOP-114875; awarded to SL), a CIHR project grant (PJT-148562; awarded to SL), a CIHR bridge grant (PJT-149092; awarded to KP/SL), a CIHR project grant (PJT-159693; awarded to KP), and by a research funding arrangement with Health Canada (#1617-HQ-000012; contract awarded to SL).

## Conflict of interest

The authors declare that the research was conducted in the absence of any commercial or financial relationships that could be construed as a potential conflict of interest.

## Publisher’s note

All claims expressed in this article are solely those of the authors and do not necessarily represent those of their affiliated organizations, or those of the publisher, the editors and the reviewers. Any product that may be evaluated in this article, or claim that may be made by its manufacturer, is not guaranteed or endorsed by the publisher.

## Footnotes

^1^ www.compass.uwaterloo.ca
